# The Effect of the Non-task Language When Trilingual People Use Two Languages in a Language Switching Experiment

**DOI:** 10.3389/fpsyg.2020.00754

**Published:** 2020-04-30

**Authors:** Jianlin Chen, Hong Liu

**Affiliations:** Department of English, School of Foreign Languages and Literatures, Lanzhou University, Lanzhou, China

**Keywords:** trilinguals, language switching, language comprehension, inhibition, task and non-task language

## Abstract

This study investigated the effect of non-task language in a language switching experiment. Non-task language refers to participants’ languages (regardless of proficiency level) that are not used in any trials throughout the experiment. We recruited 60 Tibetan-Chinese-English trilinguals (12th-grade high school students with a median age of 17) to perform a lexical decision (word vs. non-word) task in only two of their languages. We repeated the experiment three times to present each language pair once. In each experiment, the participants were divided into two groups that significantly contrasted with each other in their non-task language while remaining comparable in the two task languages. Response time (RT) and error rate (ER) have been examined to evaluate task performance. The interaction between task performance and the participants’ proficiency in the non-task language was also examined. The results showed anull effect of language switching. In addition, the effect of the non-task language was not found. These results were interpreted with reference to the main models of bilingual visual word recognition and the role of orthography specificity.

## Introduction

In this study, we investigated how Tibetan-Chinese-English trilinguals process their languages in terms of the word recognition process. Specially, we examined the effect of the non-task language, the language that is not explicitly activated for the task purpose, on a task performance involving the other two languages in a language switching experiment. By doing so, we have attempted to incorporate the processing of the non-task language and the role of orthography specificity in the existing models of bilingual word recognition, i.e., BIA+ (Dijkstra and van Heuven, 2002) and its modification BIA + s Casaponsa et al., 2019).

### Current Models on Bilingual Lexical Processing

When bilinguals are visually presented with a word, the non-selective assumption (Hermans et al., 1998; van Heuven et al., 1998; De Groot et al., 2000; Dijkstra and van Heuven, 2002; Kroll and Dijkstra, 2002; De Bot et al., 2007; Lemhöfer et al., 2008; Dijkstra et al., 2019) argues that candidates from both of their languages (including the non-target one) are activated, a phenomenon also termed “parallel activation” (Green, 1998; Costa et al., 1999; Finkbeiner et al., 2006; Philipp and Koch, 2009; Kroll et al., 2013). The non-selective activation assumption has also been argued for in the domain of language production (Dijkstra et al., 1999; von Studnitz and Green, 2002). Activation from the semantic system is spread to the lexical level, and several lexical representations are activated. The lexical selection mechanism for production is not only sensitive to the target word but also sensitive to the activation level of other non-target—but activated—words. In the meantime, some studies have extended this non-selective hypothesis in bilingual studies to trilinguals (Lemhöfer et al., 2004; Szubko-Sitarek, 2011; Kroll et al., 2013), demonstrating that when trilinguals perform a task in one language, and the other two languages are also activated.

However, the parallel activation of two or more languages very rarely causes a performance error (Gollan et al., 2011). How is a word in the target language is correctly retrieved, processed, and comprehended/produced? How are other words from the non-target languages(s) processed so as not to cause performance interference? A few models have been proposed. A major model that accounts for bilingual visual word processing is bilingual interactive activation (BIA) (Grainger and Dijkstra, 1992; Dijkstra and van Heuven, 1998) and its successor BIA+ (Dijkstra and van Heuven, 2002).

BIA and BIA+ assume parallel/non-selective bottom-up activation from letters to language. Orthographic features of the input word will activate similar letter strings from both target and non-target languages. The activated candidates will next activate their corresponding phonological and semantic representations. Identifying the input word results in the subsequent retrieval of the language membership information for that word, i.e., a language node. The inhibition of active lexical candidates is applied top-down via a language node (which reflects global lexical activity of the target language) to lexical items from the non-target language (BIA) or via adaptation of decision criteria (BIA+). However, these two models do not accommodate well the sub-lexical information, such as orthographical-specific (marked) features, which are shown to be a significant factor in visual word recognition (Grainger and Beauvillain, 1987; Thomas and Allport, 2000; Orfanidou and Sumner, 2005; Casaponsa and Duñabeitia, 2016). Some subsequent modifications of the models have been proposed, such as the BIA + extended model (BIA + d, van Kesteren et al., 2012), which adds a pre-lexical processing stage of language-specific feature-level information to speed up language attribution, and the BIA + s model (Casaponsa et al., 2019), which adds separate orthographic and phonological sub-lexical language nodes to account for language-selective effects emerging within an integrated lexicon.

With respect to bilingual lexical production, an influential model following the non-selective assumption is the Inhibition Control model (IC; Green, 1998), which argues that lexical access in bilingual speakers entails the reactive top-down inhibition of lexical items belonging to the non-target language. The asymmetrical switching cost reported by Meuter and Allport (1999) is considered to be supporting the notion that lexical access entails inhibitory processes. Another reference framework that has been frequently cited to explain empirical results in bilingual word production is the Revised Hierarchical Model (RHM; Kroll and Stewart, 1994; Kroll and Tokowicz, 2001, 2005). According to the RHM, there are shared semantics among a bilingual’s different languages, but the route to access the semantic information is different for different languages. For less proficient bilinguals, L2 is connected to concepts through prior activation of an L1 translation equivalent. Increased proficiency in L2 can strengthen the link between L2 representations and semantics, up to a certain point, and a direct link is established.

More recently, another model has been proposed to integrate bilingual visual word recognition and word translation, the Multilink Model (Dijkstra et al., 2019). Similar to the previous models, this computational model assumes language non-selective access and parallel activation of word-form neighbors. In this interactive model, an input word activates similar orthographic representations, which feed activation to their semantic and phonological counterparts, and associated language membership representations. However, no lateral inhibitory effects, either between languages or within languages, are assumed.

The models reviewed thus far, both verbal and implemented, are based on languages that share orthographical features or scripts, such as English and Dutch, or English and Spanish. The non-selective assumption is also based on the orthographic similarity between the input word and lexical candidates from both languages. However, it has already been confirmed that language-specific/marked orthographic features can speed up language attribution by reducing the number of candidates from the non-target language (van Kesteren et al., 2012; Casaponsa et al., 2019). More specifically, in Dijkstra and van Heuven (2002), it is posited that language-specific access is possible with language pairs that do not share orthography at all (e.g., Chinese and English). To successfully capture the mechanism underlying bilingual lexical processing, a comprehensive model should be able to generalize the lexical access and processing of bi-scriptal bilinguals and specify how words from orthographically distinct languages are retrieved and processed.

### Language Switching Paradigm

The models of bilingual language processing reviewed in the above section assume the role of inhibition in the control process; how inhibition is implemented and at what stage it is involved, however, vary across different models. A dominant approach to investigate the involvement of inhibition in language processing is the language switching paradigm. It is argued that switching between languages is more costly than staying in the same language [manifested as longer response time (RT) and more performance errors], and switching into the more dominant language is even more costly (Meuter and Allport, 1999; Costa and Santesteban, 2004; Costa et al., 2006). This asymmetrical pattern of switching cost, as mentioned before, has been considered the main support for the IC model and has received much empirical support in the literature, especially related to production-based switch costs (Meuter and Allport, 1999; Jackson et al., 2001; Costa and Santesteban, 2004; Philipp et al., 2007; Declerck et al., 2012; Macizo et al., 2012; Filippi et al., 2014; Slevc et al., 2016).

However, not all studies on bilingual language processing have found a switch cost or asymmetric switch cost pattern. For example, asymmetry is not always observed in production-based switch cost experiments (Hernandez and Kohnert, 1999; Costa and Santesteban, 2004; Costa et al., 2006). In the domain of language comprehension, the picture is more complex. Some studies have replicated the findings of asymmetric switch costs (e.g., Olson, 2016; Declerck and Grainger, 2017). Some other studies have also found asymmetric switch costs, but the direction was reversed, i.e., larger cost in L2 than in L1 (Proverbio et al., 2004; Chauncey et al., 2008; Bultena et al., 2015). There are also studies that found symmetrical switch costs (e.g., Macizo et al., 2012; Hirsch et al., 2015; Philipp and Huestegge, 2015). Macizo et al. (2012) argued that competition between the two activated languages was required to trigger inhibition. Several recent comprehension-based studies even failed to find switch costs (Bultena et al., 2015; Declerck and Grainger, 2017; Declerck et al., 2019; Struys et al., 2019).

Thus far, the short review has shown that findings on the language processing mechanism in comprehension tasks are inconclusive, with evidence provided both for and against the involvement of inhibition. The inconsistent findings on switch costs in both production- and comprehension-based studies calls for more solid evidence for or against the involvement of inhibition in bilingual language processes, especially when it is shown that asymmetric switching costs can be accounted for by persisting activation of the weaker language rather than persisting inhibition of the dominant language (Philipp et al., 2007). There may be different types of inhibition (e.g., see Colzato et al., 2008 for active inhibition and local reactive inhibition; see Christoffels et al., 2007 for global inhibition and local inhibition; see Declerck, 2019 for a review on proactive language control), and switch costs may not be able to assess them all (Bobb and Wodniecka, 2013; Declerck, 2019). There may be certain conditions to be satisfied before inhibition is implemented. The differences between experiments with regard to the differences in the task nature and demands, stimuli composition, and participants’ expectancies can affect these conditions.

In addition, the studies in bilingual language control share one common feature in the design of their experiment, i.e., they examine how bilinguals/multilinguals control and process the languages that are explicitly activated for task performance. A question then arises: what is the status of non-task language(s)? Non-task language is different from non-target language. Non-target languages are not the target in the current trial but will be activated afterward in a different trial. In this sense, target and non-target language(s) are explicitly activated in alternation during the experiment. Thus, we may refer to them as task languages. Non-task languages are the languages that are known by the participant but not used at all throughout the experiment. For example, when a Chinese-English-Tibetan trilingual participates in a language switch experiment where switch costs between Chinese and English are examined, Tibetan is in this case the non-task language.

Some studies on neighborhood effects suggest that when subjects perform a task in a monolingual context, e.g., performing an English lexicon decision task, their non-task language is also activated and competes with target items (van Heuven et al., 1998; Midgley et al., 2008; van Kesteren et al., 2012). Such studies are based on languages that share scripts to some extent, such as English and Spanish or English and Dutch. Wu and Thierry (2010) showed that the co-activation of the non-task language can happen to languages that do not share scripts, such as English and Chinese. The results of their experiments suggest that processing in L2 can activate native language translations, but only at the phonological level. More importantly, the accessed phonology information of the non-task native language Chinese was shown to be facilitative when the participants were asked to judge the semantic relatedness of words in L2. This finding shows that the co-activated language is not necessarily inhibited and incurs cost in processing. Therefore, the current scholarship seems to suggest that the non-task language is activated at some level, but the evidence on how it interacts with the task performance is inconclusive.

## Current Study

The primary focus of the present study is on the non-task language effects in language switching experiments where only two of the trilingual participants’ languages are explicitly activated for the task performance. We explore whether the non-task language will be processed in a manner that might affect the switching performance of the two task languages. The current scholarship on language processing mechanism informs us little on this question. However, knowing how the non-task language is processed not only provides further insights into the involvement of inhibition in language processing but also contributes to a more comprehensive theory on language control mechanism.

In addition, we have attempted to provide some insight into the effects of orthographic specificity in language switching and language processing in general. We conducted three experiments where trilinguals with three orthographically and phonologically different languages (i.e., Tibetan, Chinese, and English) performed a comprehension-based language switching task—a generalized lexical decision task using only two of their languages in each experiment. The participants were required to respond with “yes” to words (of either language) and with “no” to non-words (Dijkstra et al., 1998; van Heuven et al., 1998; Lemhöfer and Dijkstra, 2004). The design of the present experiments has been specified in the following sections.

According to the BIA + model or its modifications (Dijkstra and van Heuven, 2002; van Kesteren et al., 2012; Casaponsa et al., 2019), bilinguals’ languages should be simultaneously activated at the orthographical and/or phonological level, regardless of whether or not they are the target. Therefore, the non-target language not used in the current trial and the non-task language not explicitly used at all in the experiment should both be activated and exert some effects on the task performance. However, previous studies have shown that orthographic specificity can reduce switching effects (Grainger and Beauvillain, 1987; Orfanidou and Sumner, 2005; although see Thomas and Allport, 2000). By investigating whether and how the effects of orthographic markedness may emerge in a language switching experiment where both the non-target and the non-task language are examined, the present study made a tentative attempt to test whether and how the BIA + model or its modifications can be generalized to explain the experiment results involving orthographically and phonologically different languages.

## Experiment 1

### Methods

The participants performed a generalized lexical decision task, i.e., words or non-words, where they made yes/no response to the visual presentations of words and non-words from both languages (L1 and L2 in Experiment 1, L1 and L3 in Experiment 2, and L2 and L3 in Experiment 3). The letter strings or the characters activate orthographic, semantic, and phonological codes, which help the participants discriminate between word and non-word input. The order of the experiments was counterbalanced for participants. The experiments were conducted within the same day, however, with an interval of a few classes (including English, Chinese, and Tibetan classes).

#### Participants

The participants were recruited from one of the secondary schools reserved for Tibetan students in the northwest region of mainland China. The reason we chose high school students as our participants, instead of college students as other studies have been doing, was because it was much less likely that we could collect a sufficient number of comparable Tibetan-Chinese-English participants in other places than those reserved school for Tibetan students. The present study did not interfere with the participants’ classes. We collected data at the end of the semester and all participants gave written informed consent for data collection in accordance with the Declaration of Helsinki.

There are two types of curricula in those schools: the Tibetan-mediated curriculum (TMC) and the Chinese-mediated curriculum (CMC). Tibetan is the language of instruction in the TMC, and Chinese in the CMC. Sixty 12th-grade students [with a median age of 17 (*SD* = 0.8)], 30 from the TMC and 30 from the CMC, participated in the experiments. A language history questionnaire adopted from Chen (2018) was used to investigate the participants’ language-learning background. Their language proficiency was self-reported on a seven-point scale (1 = lowest proficiency, 7 = highest proficiency). The self-report was administered by their respective teachers and then subjected to their teachers’ confirmation of reliability. The results are shown in Table 1. Most of the students from both curriculum types began to learn Chinese as a second language in Grade 1 (about 6 years old, with an exposure of about 11 years or more), or even earlier, and English as a third language in Grade 3 (about 9 years old, with an exposure of about 8 years). Therefore, for these students, Tibetan is their L1, Chinese L2, and English their L3.

**TABLE 1:** Language history information (mean and standard deviation) of the participants.

**Participant groups**	**Language**	**Age of acquisition**	**Proficiency**	**Use in different domains (%)**	
				**School**	**Home**
TMC	L1	1.9 (1.9)	5.87 (0.87)	79.4 (10.7)	92.6 (12.3)
	L2	5.3 (1.4)	4.90 (0.96)	15.0 (07.1)	06.7 (11.7)
	L3	9.1 (1.0)	3.57 (0.92)	05.6 (04.2)	00.7 (02.1)
CMC	L1	3.3 (3.4)	5.57 (0.97)	44.9 (17.0)	90.1 (07.5)
	L2	4.9 (1.9)	5.77 (0.77)	49.7 (18.0)	09.6 (07.6)
	L3	8.9 (1.2)	3.17 (1.09)	05.4 (05.0)	00.3 (00.9)

However, within each group, their relative proficiency in L1 and L2 differed. For the TMC students, they were most proficient in their L1 Tibetan, as indicated by the significant difference between L1 and L2, *t* = 4.52, *p* < 0.001, and between L1 and L3, *t* = 10.45, *p* < 0.001. Their L2 Chinese was also significantly more proficient than L3 English (*t* = 5.79, *p* < 0.001). As for the CMC students, there were significant differences between their L1 and L3 (*t* = 9.05, *p* < 0.001), and between L2 and L3 (*t* = 10.76, *p* < 0.001). However, there was no significant difference between their L1 and L2 (*t* = −0.89, *p* = 0.38). Between the two groups, they were comparable in L1 (t = 1.32, *p* = 0.19) and L3 (t = 1.55, *p* = 0.13), but they significantly differed in L2 (t = −4.1, *p* < 0.01). However, it should be noted that both groups can be considered overall proficient in their L1 and L2, as indexed by the mean values of 4.9 or above for both languages reported by both groups.

In terms of language use, the TMC students used Tibetan much more frequently than the other two languages both in school and at home. The CMC students used Tibetan usually at home but used Chinese for nearly half of the time in school. Their exposure to and degree of use of L2 English was greater than that of the TMC students. For both groups of students, English use was confined to classroom settings.

In order to test whether the participants’ proficiency in the non-task language interacted with their task performance in the experiments, for each experiment, we split the participants by mean into the higher and lower non-task language proficiency groups who contrasted significantly only in their non-task language and remained comparable in the task languages. Therefore, for Experiment 1, the participants were divided into the higher L3 group (*N* = 26, *M* = 4.42, *SD* = 0.57) and lower L3 group (*N* = 24, *M* = 2.75, *SD* = 0.63). Ten participants had to be removed because their inclusion would result in a significant difference between the higher and lower L3 groups on their L2 or L1. The difference between these two groups on L3 proficiency was significant (*t* = 10.45, *p* < *0.001*). There was no significant difference in L1 proficiency between the two groups, M (L3 higher) = 6.08 (0.73), M (L3 lower) = 5.88 (1.05), *t* = 0.77, *p* = 0.45, nor in their L2, M (L3 higher) = 5.81 (0.79), M (L3 lower) = 5.71 (0.73), *t* = 0.41, *p* = 0.69.

#### Task and Stimuli

The participants were asked to perform the generalized lexical decision task in their L1 (Tibetan) and L2 (Chinese). There were three experimental blocks in the experiment, and each block consisted of 20 trials in each language, 10 word trials and 10 non-word trials. Each language consisted of 50% switch trials and 50% repetition trials. The sequence of trials was randomized for each participant group. Each participant needed to perform 30 word trials and 30 non-word trials per language in each experiment.

The stimuli pool of the three experiments consisted of 60 words and 60 non-words for each language. The selection of the stimuli words was as follows. A total of 120 L2 words were selected from The Contemporary Chinese Dictionary. These words were comparable in terms of strokes, with an average number of 15.52 strokes (*SD* = 7.62). Ten peer students who did not attend the experiments rated the familiarity of these words using a seven-point scale (1 = least familiar, 7 = most familiar). Results showed that the participants were all familiar with the L2 words (with a mean score of 6.58). A total of 60 of these words were used as word stimuli (30 in experiment 1 and 30 in experiment 3), and the other 60 were used to create L2 non-words for experiments 1 and 3 by replacing one character in two-/three-character Chinese words with a homonym to make the word meaningless. For example, the non-word 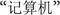 was created by replacing the first character 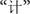 of the word 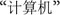 (computer) with its homonym 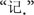


**FIGURE 1:**
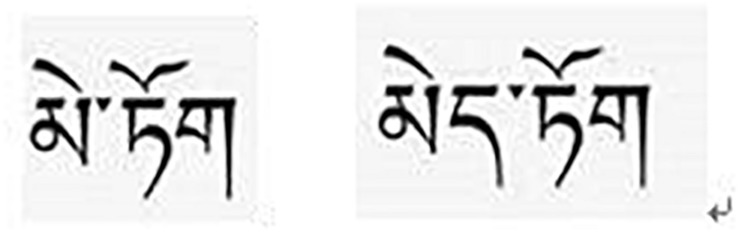
An example of a word and a non-word in Tibetan, meaning “flower” (the left is a word and the right is a non-word).

Another 120 L2 words that were not used as word stimuli, but they were comparable to stimuli L2 words in terms of word familiarity were also selected and translated by a local Tibetan-Chinese bilingual teacher to L1 Tibetan equivalents. The Tibetan equivalents were also rated for similarity by these 10 peer students. The results showed that they were familiar with them (with a mean score of 6.71). The average number of syllabus of these words is 2.1 (*SD* = 0.83). Sixty of these Tibetan words were used as word stimuli (30 in experiment 1 and 30 in experiment 2), and the rest were used to create L1 non-words for experiments 1 and 2 by deleting or adding a vowel or a consonant to a word to make it meaningless (see Figure 1).

A total of 120 L3 English words, which were comparable in terms of syllabus (with a median of 1.74 syllabus, *SD* = 0.71), were selected from the English textbook used in the junior Tibetan schools (Grade 7–9) and subjected to familiarity test. The results showed a high level of familiarity (mean score: 6.43). Similarly, 60 of these English words were used as stimuli words (30 in experiment 2 and 30 in experiment 3), and the rest were used to create non-words for experiments 2 and 3 by deleting or adding a letter in a legitimate word (e.g., wrd and booy).

Overall, the participants’ familiarity with the chosen words from the three languages did not differ significantly (*F* (1, 118) = 0.801, *p* = 0.372). The complete stimuli lists for experiments 1, 2, and 3 are provided in Supplementary Appendices A–C, respectively. It should be noted that non-words in the three languages were created in different ways. With L2 Chinese, the non-words are pronounceable but meaningless. However, the characters that make up the non-words are legitimate characters in Chinese. With L1 Tibetan, the non-words are meaningless and illegitimate in Tibetan, but they are pronounceable. As for L3 English, the non-words are meaningless, illegitimate, and unpronounceable^1^.

#### Procedure

Prior to the experiment, the instructions were presented both orally and visually. The participants were told that a series of letter strings/characters would appear on the screen, one after the other, and that they had to decide as quickly and as accurately as possible whether each of the presented items was a word (Tibetan or Chinese) or not. Following the instructions, the participants performed a practice block of six trials. Stimuli were visually presented one by one for 3000 ms in the center of the computer screen with black text on a white background. The participants indicated their decision by pressing the right SHIFT key for word and the left SHIFT key for non-word (the mapping of the response keys to either decision was counterbalanced across participants). Stimuli stayed visible during the 3000 ms duration or until a response was registered. After the participant’s response there was a 600 ms interval until the next stimulus would be presented.

#### Analysis and Results

For the three experiments reported in this paper, participants’ RTs and error rates (ERs), which were recorded by DMDX software (Forster and Davis, 1984), were analyzed. Error trials were excluded from RT analyses. RTs and ERs on non-words were not included in the current analyses. In addition, RT above or below three standard deviations from the mean (per participant) were deleted. Data of the participants whose ERs were above 50% were also deleted. Taking these criteria into account resulted in the exclusion of 4.7% of the data. The mean RTs and ERs in Experiment 1 for different conditions and groups are shown in Table 2.

**TABLE 2:** RTs and ERs in experiment 1.

	**Mean RT (SD) in ms**				**Mean ER (SD) (%)**			
	**Switch**		**Non-switch**		**Switch**		**Non-switch**	
	**To L1**	**To L2**	**To L1**	**To L2**	**To L1**	**To L2**	**To L1**	**To L2**
Higher L3	903.03 (228.33)	858.76 (238.28)	825.62 (223.74)	813.43 (213.93)	5.34 (8.27)	2.29 (4.21)	4.65 (8.20)	2.88 (5.24)
Lower L3	897.53 (240.21)	839.13 (218.02)	881.67 (234.18)	834.14 (212.64)	7.27 (8.59)	3.67 (6.30)	8.94 (10.74)	1.82 (4.83)

For the analysis of RTs, we used linear mixed-effect (LMM) models with items and participants as random effects (Baayen, 2008). As fixed effects, we entered language (Experiment 1: Chinese as reference vs. Tibetan; Experiment 2: English as reference vs. Tibetan; Experiment 3: Chinese as reference vs. English), trial type (switch vs. non-switch as reference), and proficiency in the non-task language (high as reference vs. low) as well as the interaction terms. The models were fitted with the lmer function from the lme4 package (Bates et al., 2015) in the R statistical computing environment. Regression coefficients (b), standard errors (SE), and *t*-values for significant factors and interactions were reported. Fixed effects were considered reliable if | *t*| > 1.96 (Baayen, 2008). To analyze ERs, we fitted the logistic mixed models (Jaeger, 2008) with the glmer function in the R environment, again with items and participants as random effects and the same set of fixed factors and their interaction terms. Here, instead of reporting *t*-values, we reported *z*-values, which can be interrupted in the same way as the *t*-values.

**FIGURE 2:**
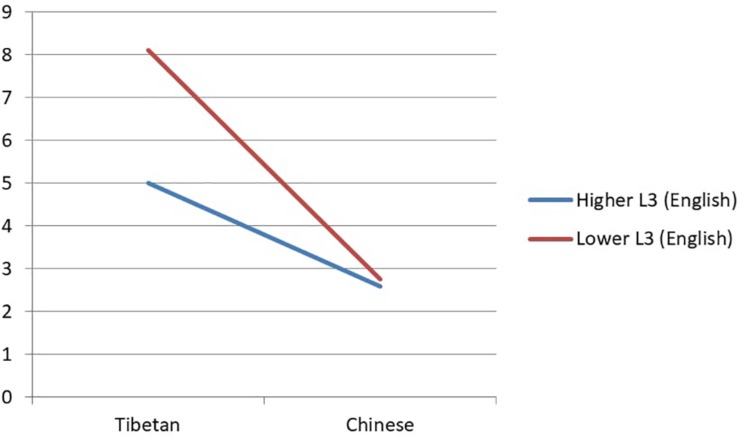
The interaction between language and the non-task language proficiency in ERs in Experiment 2.

In the analyses of RTs, the results showed no main effect of any individual factors. No significant interaction was found. In the analyses of ERs, there was a marginally significant interaction between language and proficiency in the non-task language (Tibetan: low L3: *b* = −1.30, *SE* = 0.73, *z* = −1.97). The high and low L3 groups performed similarly in Chinese. The high L3 group had an ER of 2.59% and the low L3 group an ER of 2.75%. However, the low L3 group made more mistakes in Tibetan (8.12%) while the high group made less (5.00%). The interaction is shown in Figure 2.

#### Discussion

In Experiment 1, no effect of trial type was found. This means that the participants’ performance in making their lexical decision in the current trial was not affected significantly by whether the previous trial was in a different language or in the same language, thus resulting in no switch cost. A possible account for this result is to assume that the participants had language-specific access to their languages (Tibetan and Chinese). The BIA + model and its successors claim that when an input letter strings/characters are presented to bilinguals, orthographic representations that are similar to the input word regardless of language membership are activated (Grainger and Dijkstra, 1992; Dijkstra and van Heuven, 1998, 2002; van Kesteren et al., 2012). If the two languages differ with respect to their input codes, the activated set of neighbors may become much smaller. For languages that do not share input codes at all, the lexical items from the non-target language should not be activated at all, at least at the level of orthographic representation. The activation of the orthographic representation will next activate the corresponding semantic representation and/or phonological representation. The activation of the semantic representation can further feed activation to corresponding phonological representations of both languages (Casaponsa et al., 2019). However, the activation of phonological representations from the non-target language does not seem to adversely affect the lexical decision in the target language as the trial type did not significantly influence the task performance.

The interaction between language and non-task language proficiency in ERs suggested that the lower L3 group made more mistakes in Tibetan, whereas non-task language proficiency did not affect the task performance in Chinese. Here, there were two questions worth asking. One is why the non-task language affected task performance while the switching between the task languages did not. The other is why task performance in Tibetan and not in Chinese was affected. To answer these two questions, we needed to analyze the typological features of the three languages first. The three languages under discussion are orthographically and phonologically different. However, compared to Chinese, which features no correspondence between its character and pronunciation, Tibetan is closer to English in that there is a sort of mapping between spelling and pronunciation, despite the fact that English is an alphabetic language whereas Tibetan uses a syllabary. Therefore, it is possible that an improved proficiency in English means the participants are better at mapping pronunciation to spelling and vice versa. This improved ability can be carried over to Tibetan in that the orthographic input can be more quickly mapped onto the existing phonological representation (cf. Wu and Thierry, 2010). This can in turn facilitate lexical decision as orthographic input is mapped twice onto existing mental lexicon, once through semantic representation and once through phonological representation. This may explain why lexical decision in Tibetan was more accurate with the participants more proficient in English. Because Chinese is opaque in terms of the correspondence between its characters and pronunciation, the participants’ performance in the Chinese lexical decision would not benefit from their proficiency in the non-task language English.

## Experiment 2

### Methods

#### Participants

The participants were divided into a higher L2 group (*N* = 28, *M* = 6.36, *SD* = 0.48) and lower L2 group (*N* = 30, *M* = 4.73, *SD* = 0.44). Two participants had to be removed because their inclusion would result in a significant difference between the higher and lower L2 groups on their L1 or L3. The difference between these two groups on L2 proficiency was significant (*t* = 13.15, *p* < *0.001*). There was no significant difference in L1 proficiency between the two groups, M (L2 higher) = 6.04 (0.91), M (L2 lower) = 5.80 (0.87), *t* = 0.99, *p* = 0.33, nor in their L3, M (L2 higher) = 3.57 (1.24), M (L3 lower) = 3.40 (0.77), *t* = 0.62, *p* = 0.54.

#### Task and Stimuli

The participants performed the same lexical decision task in their L1 (Tibetan) and L3 (English). The stimuli words for L1 (Tibetan) and L3 (English) were drawn from the pool as described in Experiment 1.

#### Procedure

The procedure of Experiment 2 was the same as that of Experiment 1.

#### Analysis and Results

The analytical methods of Experiment 2 data were the same as those reported for Experiment 1. The mean RTs and ERs in Experiment 2 for different conditions and groups are shown in Table 3. In the analyses of RTs, the results showed no main effect of any individual factors. No significant interaction was found. In the analyses of Ers, the main effect of language was significant (*d* = −2.11, *SE* = 0.71, *z* = −2.97). Words in English were identified with more accuracy (Ers = 12.20%) compared with Tibetan words (Ers = 8.05%). No significant interaction effects were identified.

**TABLE 3:** RTs and ERs in experiment 2.

	**Mean RT (SD) in ms**				**Mean ER (SD) (%)**			
	**Switch**		**Non-switch**		**Switch**		**Non-switch**	
	**To L1**	**To L3**	**To L1**	**To L3**	**To L1**	**To L3**	**To L1**	**To L3**
Higher L2	865.82 (230.05)	867.87 (204.59)	838.22 (254.52)	826.65 (237.18)	10.64 (14.05)	5.36 (8.79)	18.83 (13.27)	4.62 (6.60)
Lower L2	917.73 (247.88)	907.66 (220.10)	844.71 (217.93)	834.41 (227.19)	6.23 (9.57)	4.41 (9.50)	13.11 (10.98)	4.97 (8.23)

#### Discussion

Much like Experiment 1, no effect of trial type was found in Experiment 2. Unlike the results of Experiment 1, the effect of non-task language proficiency in its interaction with other factors disappeared. The only significant effect was that of language. The effect of language has been taken up in the general discussion.

As argued in Experiment 1, the lexical decision depends on the mapping of the orthographic input onto the existing lexicon. The difference between the task languages, Tibetan and English, in terms of their marked orthographic representations, enables language-specific access. Therefore, no lexical candidates from the non-target language are activated at the orthographical level when the input word is visually presented. The language switching did not produce an effect on the task performance. The disappearance of the effect of the non-task language in this experiment was expected. We argued that, for languages that feature a certain level of correspondence between spelling and pronunciation, the lexical decision process may be implemented by mapping the orthographic input onto the existing mental lexicon via both semantic and phonological routes. The ability of mapping orthographic representation onto phonological representation can be enhanced if the languages of the participants are similar in terms of this spelling/pronunciation correspondence. We used this to account for the facilitative effects of English on Tibetan words decision. In a similar vein, if one of the participants’ languages does not host this spelling/pronunciation correspondence, their proficiency in this language should be of no help in making lexical decisions in languages that do. This is what the results of Experiment 2 showed. It should be noted that this facilitation effect discussed between languages that do not share script but feature spelling/pronunciation correspondence do not contradict the null effect of language switching, as the facilitative effect can be mutual and thus are canceled out.

## Experiment 3

### Methods

#### Participants

The participants were divided into the higher L1 group (*N* = 38, *M* = 6.47, *SD* = 0.50) and lower L2 group (*N* = 20, *M* = 4.85, *SD* = 0.36)^2^. Two participants had to be removed because their inclusion would result in a significant difference between the higher and lower L1 groups on their L2 or L3. The difference between these two groups on L1 proficiency was significant (*t* = 14.00, *p* < *0.001*). There was no significant difference in L2 proficiency between the two groups, M (L1 higher) = 5.61 (0.87), M (L1 lower) = 5.35 (1.01), *t* = 0.93, *p* = 0.36, nor in their L3, M (L1 higher) = 3.53 (1.09), M (L1 lower) = 3.55 (0.86), *t* = 0.47, *p* = 0.64.

#### Task and Stimuli

The participants performed the same lexical decision task in their L2 (Chinese) and L3 (English). The stimuli words for L2 (Chinese) and L3 (English) were drawn from the pool as described in Experiment 1.

#### Procedure

The procedure of Experiment 3 was the same as that of Experiments 1 and 2.

#### Analysis and Results

The mean RTs and ERs in Experiment 3 for different conditions and groups are shown in Table 4. In the analyses of RTs, the results showed that the only significant effect was that of language (*d* = 145.89, *SE* = 61.52, *t* = 2.37). The participants were faster at Chinese trials (780.96 ms) compared with English trials (908.35 ms). No significant interactions were found. In the analyses of ERs, there was no main significant effect nor interactions identified.

**TABLE 4:** RTs and ERs in experiment 3.

	**Mean RT (SD) in ms**				**Mean ER (SD) (%)**			
	**Switch**		**Non-switch**		**Switch**		**Non-switch**	
	**To L2**	**To L3**	**To L2**	**To L3**	**To L2**	**To L3**	**To L2**	**To L3**
Higher L1	814.67 (234.32)	957.15 (234.84)	758.09 (213.48)	889.30 (219.75)	4.13 (6.20)	7.38 (11.82)	2.53 (4.91)	6.81 (8.32)
Lower L1	805.75 (247.00)	913.97 (258.49)	725.27 (213.28)	824.55 (232.65)	2.28 (4.21)	5.53 (6.91)	0.00 (0.00)	7.05 (6.38)

#### Discussion

Similar to the previous two experiments, there was no effect of trial type in Experiment 3. The effect of language will be taken up in the general discussion. In this experiment, the two task languages were L2 Chinese and L3 English. As argued above, the relative proficiency in L1 Tibetan should cause performance difference in L3 English. However, the effect of the non-task language proficiency was not significant nor was any interaction involving this factor. A possible reason could be that the difference in L1 proficiency between the high and low groups was too small to lead to any effects of or interactions with the non-task language proficiency. After all, this non-task language was the first-acquired and the dominant language for both balanced and unbalanced groups. The difference self-reported by the participants when prompted, though significant, may have exaggerated the real difference that lay between the two groups.

## General Discussion

The primary focus of this study is on the effect of the non-task language of trilinguals when only two of their languages were explicitly involved in language switching experiment. The subjects participated in three experiments where they performed a generalized lexical decision task in each experiment. We controlled the participants’ proficiency in the task languages and examined how the higher and lower non-task language proficient groups may differ in their task performance.

Across the three experiments, only Experiment 1 showed that there was an interaction between the participants’ non-task language performance and language, suggesting that the participants who were more proficient in English made fewer mistakes when making lexical decisions in Tibetan but not in Chinese. We explained this as a carry-over effect of an improved ability in spelling/orthographic mapping, which can speed up the mapping of the orthographic input onto existing mental lexicon by providing double route (semantic and phonological representations). However, the facilitative effects of English on Tibetan word identification do not necessarily mean that the participants processed the supposedly activated language in a way that can facilitate the task performance. It is possible that this carry-over effect of an improved ability in spelling/orthographic mapping can function independently from the activated status of the non-task language. In this sense, the non-task language can be activated, at least at the phonological level but does not interact with task performance.

On the other hand, the results of the experiments did not show any significant influence from language switching, which indicates that the participants performed with similar RTs and ERs regardless of the language of the previous trial. A Bayesian analysis of the null hypothesis (i.e., RTs were not influenced by trial type, i.e., repetition vs. switch) was carried out across the experiments (Rouder et al., 2009). We found evidence for the main effect of experiment (Bayes factor: 687,681: 1). But there was no clear evidence of the main effect of trial type (Bayes factor: 1.99:1) nor was there clear evidence for or against an interaction between trial type and experiment (Bayes factor: 1.73:1) (Kass and Raftery, 1995).

Based on the assumptions of the BIA + model (Dijkstra and van Heuven, 2002), bilinguals’ languages are co-activated when they need to perform a task in one language only, and the co-activation takes place at the orthographic level. The null effect of language switching in the present study does not support this assumption. If both languages were activated at the orthographic level, we should witness a processing cost when the participants switched languages compared to remaining in the same language. This is because the recognition of a word in the target language will lead to inhibition of non-target orthographic lexical representations. At switch trials, processing of previously non-target items would then require overcoming the inhibition implemented at the previous trial, thus delaying lexical selection and subsequent processing. However, switch cost was not shown in any of the experiment. The explanation we propose is that our Tibetan-Chinese-English trilinguals have language-specific access to their languages because of the differences between their languages in orthography and phonology. The visual presentation of the input word/character does not activate orthographic representation in languages other than the target one, as there is no similarity in script. When there is no competitor from other languages activated, there is no need to implement inhibition (Macizo et al., 2012). Previous studies have also reported similar findings that switch costs can be mitigated or even eliminated by the presence of language-specific orthographic cues (Grainger and Beauvillain, 1987; Orfanidou and Sumner, 2005; although see Thomas and Allport, 2000).

The BIA + d model (van Kesteren et al., 2012) and the BIA + s model (Casaponsa et al., 2019) both consider the encoding of language-specific orthographical features in the existing BIA + model and argue that the language-specific (marked) features of orthography can help subjects to gain a language decision advantage at a pre-lexical processing stage as these features can set the lexical candidates to one language and lead to faster recognition of language-specific words as a result of less competition. However, the current results are better accounted for by the BIA + s model compared to the BIA + d model. In BIA + d, it is suggested that language-specific orthographic features can give speakers an identification advantage in language decision task but not in lexical decision tasks, as lexical access cannot be restricted to words of the target language only. If this were the case in the present study, we should have observed some form of switch costs. The BIA + s model, on the other hand, contains inhibitory links between orthographic sub-lexical language nodes and their corresponding lexical forms. Such inhibitory links can prevent the forms of the non-target language from being activated at the lexical level after language membership has been identified at the pre-lexical level, and thus allow language-selective effects to emerge. The implementation of such links can predict the null effect of language switch in the current study. Therefore, though the BIA + s model is built on the evidence collected from languages that contain marked orthographic features but still share scripts, it seems that it can also be generalized to languages that do not share scripts.

However, according to BIA + s, cross-language activation inhibited at the orthographical level as a result of language-specific access does not mean that only the target language is activated. The non-target phonological representations can be activated by receiving activation from the shared and activated semantics (Kroll and Stewart, 1994), even in the case where the two languages do not share scripts (Wu and Thierry, 2010). The BIA + s model implements a separate phonological sub-lexical language node in addition to a separate orthographic language node. According to BIA + s, cross-language activation that is inhibited by marked orthographic lexical representations can be derived from phonological lexical representations via the mediation of semantics. However, the activation in the phonological lexical representation of the non-task language does not interact with task performance in the present lexical decision task, similarly to the non-target language.

Having now returned to the primary concern of the current study, we have asked, “How is the non-task language processed?” So far, it seems that the examination of the non-target and the non-task language has shown similar results on task performance in the language switching experiment. We may take this as evidence to propose that, in trilingual word recognition, the non-target and the non-task language are processed in a similar way, that is, they are both treated as task irrelevant, regardless of being artificially activated for task purpose.

Therefore, we can draw a tentative conclusion that, when a speaker is presented a visual word, not only the target language items will be activated, the items from the task irrelevant languages, including both the non-target and the non-task, are activated too. This applies to languages that do not share scripts as well. However, this co-activation of languages that do not share scripts is different from the non-selective language access assumption in the current main models of bilingual visual word recognition, which argues for the activation of similar orthographic forms from both languages. The co-activation may only take place at the phonological level and the activated non-target phonological activation does not need inhibition in the lexical decision task where the response is made based on the mapping between orthographical representations.

Finally, language has emerged as a significant factor in ERs or RTs across the three experiments. However, it is difficult to assess the effect of this factor in the current study. As explained in the method section of Experiment 1, the non-words of each language were formed in a different way. With L2 Chinese, the non-words are pronounceable but meaningless. The characters that make up the non-words are legitimate characters in Chinese. With L1 Tibetan, the non-words are similarly pronounceable, but meaningless and illegitimate in Tibetan. As for L3 English, the non-words are meaningless, illegitimate, and unpronounceable. From the perspective of the participants, the task may be easier in English as the discrimination of words from non-words may be more straightforward. For this reason, different RTs and ERs in each language across the experiments cannot be simply compared to argue for the role of language proficiency.

## Conclusion

The present study investigated the effect of the non-task language in a language switching experiment where the Tibetan-Chinese-English trilinguals were asked to perform a generalized lexical decision task in two of their languages. In addition to the null effect of language switching, the results did not show any significant non-task language effect. We have proposed that languages that are not used in the current generalized lexical decision task, the non-target and the non-task language, are processed in a similar way. By taking into account the orthographic specificity of the three languages involved, and have we suggested that the absence of switch cost and the null effect of the non-task language can be explained by the BIA + s model (a modification of the BIA + model). The findings of the present study have provided empirical evidence to support the generalizability of the BIA + s model to languages that are orthographically and phonologically different.

However, the settings of the experiments and the nature of the task may have contributed to the current results, especially the null effect of the non-task language. Future research can carry out extensions of the current study with some production-based tasks, such as picture-naming, and/or production-comprehension combined tasks, such as word translation. Using production-based tasks, during which languages are activated in a top-down manner, can provide further insights into the language processing mechanism of multilinguals. At the same time, the current study has suffered from two main limitations in methods, which also point to the direction for future research. One limitation is the use of subjective self-ratings to measure language proficiency. Though widely used in the field to test language proficiency, self-reports can sometimes be problematic (Lemhöfer and Broersma, 2012). We included the teachers’ comments to offset the potential subjective bias. However, we cannot completely exclude the possibility that some students may have under-/over-estimated their language proficiency in one or more languages. Future studies should address this limitation by combining both subjective self-rating and objective language test. Another limitation is that the three experiments were carried out within the same day, which may have artificially increased the activation level of the three languages, especially among participants who were less proficient in any one of the languages. Future studies should try to avoid such an effect through either recruiting different participants for the three experiments or performing the three experiments on different days.

## Data Availability Statement

The datasets generated for this study are available on request to the corresponding author.

## Ethics Statement

Ethical review and approval was not required for the study on human participants in accordance with the local legislation and institutional requirements. Written informed consent to participate in this study was provided by the participants’ legal guardian/next of kin.

## Author Contributions

JC was responsible for research design, data collection and coding, and contributed to manuscript writing. HL was responsible for data analyses and manuscript writing, and contributed to data coding. Both authors were responsible for the results and the interpretation of the results.

## Conflict of Interest

The authors declare that the research was conducted in the absence of any commercial or financial relationships that could be construed as a potential conflict of interest.

## Footnotes

1 We are aware that the way we created English non-words may appear simple. In some previous studies, English non-words were constructed by replacing one vowel or the first consonant of a real word with a different one to make the word illegitimate (e.g., Cui and Zhang, 2009; Mosca and de Bot, 2017). However, the participants in these studies were either college students or moderately proficient English learners. The present study recruited secondary school students who are proficient in Tibetan and/or Chinese but much less proficient in English. Therefore, they may not be sensitive enough to the formation rules of English words and hence not may be able to perform the task in a meaningful way. For this reason, our English non-words were created in a simpler way.

2 Though our participants have Tibetan as their L1, it does not necessarily mean that they are equally proficient in their language skills, especially in writing and/or reading. This may be especially the case for the CMC students, who had Tibetan only as a course instead of the instruction language in school. This was reflected in the students’ language self-report, which made it possible for the division of the higher and lower L1 groups.

## References

[B1] Baayen R. (2008). *Analyzing Linguistic Data: A Practical Introduction to Statistics.* Cambridge: Cambridge University Press.

[B2] Bates D. Mächler M. Bolker B. Walker S. (2015). Fitting linear mixed-effects models using lme4. *J. Stat. Soft.* 67 1–48. 10.18637/jss.v067.i01

[B3] Bobb S. Wodniecka Z. (2013). Language switching in picture naming: what asymmetric switch costs (do not) tell us about inhibition in bilingual speech planning. *J. Cogn. Psychol.* 25 568–585. 10.1080/20445911.2013.792822

[B4] Bultena S. Dijkstra T. van Hell J. G. (2015). Language switch costs in sentence comprehension depend on language dominance: evidence from self-paced reading. *Biling. Lang. Cogn.* 18 453–469. 10.1017/S1366728914000145

[B5] Casaponsa A. Duñabeitia J. A. (2016). Lexical organization of language-ambiguous and language-specific words in bilinguals. *Q. J. Exp. Psychol.* 69 589–604. 10.1080/17470218.2015.1064977 26123205

[B6] Casaponsa A. Thierry G. Duñabeitia J. A. (2019). The role of orthotactics in language switching: an ERP investigation using masked language priming. *Brain Sci.* 10:22. 10.3390/brainsci10010022 31906199 PMC7016794

[B7] Chauncey K. Grainger J. Holcomb P. J. (2008). Code-switching effects in bilingual word recognition: a masked priming study with event-related potentials. *Brain Lang.* 105 161–174. 10.1016/j.bandl.2007.11.006 18191445 PMC2684792

[B8] Chen J. (2018). How do L3 words find conceptual parasitic hosts in typologically distant L1 or L2? evidence from a cross-linguistic priming effect. *Int. J. Biling. Edu. Biling.* 10.1080/13670050.2018.1439879

[B9] Christoffels I. K. Firk C. Schiller N. O. (2007). Bilingual language control: an event-related brain potential study. *Brain Res.* 1147 192–208. 10.1016/j.brainres.2007.01.137 17391649

[B10] Colzato L. S. Bajo M. T. van den Wildenberg W. Paolieri D. Nieuwenhuis S. T. La Heij W. (2008). How does Biling. improve executive control? A comparison of active and reactive inhibition mechanisms. *J. Exp. Psychol.* 34 302–312. 10.1037/0278-7393.34.2.302 18315407

[B11] Costa A. Miozzo M. Caramazza A. (1999). Lexical selection in bilinguals: Do words in the bilingual’s two lexicons compete for selection? *J. Mem. Lang.* 41 365–397. 10.1006/jmla.1999.2651

[B12] Costa A. Santesteban M. (2004). Lexical access in bilingual speech production: evidence from language switching in high-proficient bilinguals and L2 learners. *J. Mem. Lang.* 50 491–511. 10.1016/j.jml.2004.02.002

[B13] Costa A. Santesteban M. Ivanova I. (2006). How do highly proficient bilinguals control their lexicalization process? Inhibitory and language-specific selection mechanisms are both functional. *J. Exp. Psychol. Learn. Mem. Cogn.* 32 1057–1074. 16938046 10.1037/0278-7393.32.5.1057

[B14] Cui Z. Zhang J. (2009). Linguistic association model for tibetan-mandarin English Trilingual. *Acta Psychol. Sin.* 41 208–219.

[B15] De Bot K. Lowie W. Verspoor M. (2007). A dynamic systems theory approach to second language acquisition. *Biling. Lang. Cogn.* 10 7–21.

[B16] De Groot A. M. Delmaar B. P. Lupker S. J. (2000). The processing of interlexical homographs in translation recognition and lexical decision: support for non-selective access to bilingual memory. *Q. J. Exp. Psychol. Sect. A* 53 397–428. 10881612 10.1080/713755891

[B17] Declerck M. (2019). What about proactive language control? *Psychon. Bull. Rev.* 27 24–35. 10.3758/s13423-019-01654-1 PMC7000488 31410740

[B18] Declerck M. Grainger J. (2017). Inducing asymmetrical switch costs in bilingual language comprehension by language practice. *Acta Psychol.* 178 100–106. 10.1016/j.actpsy.2017.06.002 28646654

[B19] Declerck M. Koch I. Duñabeitia J. A. Grainger J. Stephan D. N. (2019). What absent switch costs and mixing costs during bilingual language comprehension can tell us about language control. *J. Exp. Psychol. Hum. Percept. Perform.* 45 771–789. 10.1037/xhp0000627 30920253

[B20] Declerck M. Koch I. Philipp A. M. (2012). Digits vs. Pictures: the influence of stimulus type on language switching. *Biling. Lang. Cogn.* 15 896–904.

[B21] Dijkstra T. Grainger J. van Heuven W. J. (1999). Recognition of cognates and interlingual homographs: the neglected role of phonology. *J. Mem. Lang.* 41 496–518.

[B22] Dijkstra T. van Heuven W. J. B. (1998). “The BIA model and bilingual word recognition,” in *Localist Connectionist Approaches to Human Cognition*, eds Grainger J. Jacobs A. M. (Mahwah, NJ: Lawrence Erlbaum Associates), 189–225.

[B23] Dijkstra T. van Heuven W. J. B. (2002). The architecture of the bilingual word recognition system: from indentification to decision. *Biling. Lang. Cogn.* 5 175–197. 15905078

[B24] Dijkstra T. van Heuven W. J. B. Grainger J. (1998). Simulating competitor effects with the Bilingual Interactive Activation Model. *Psychol. Belg.* 38 177–196.

[B25] Dijkstra T. Wahl A. Buytenhuijs F. Halem N. V. Al-Jibouri Z. de Korte M. (2019). Multilink: a computational model for bilingual word recognition and word translation. *Biling. Lang. Cogn.* 22 657–679.

[B26] Filippi R. Karaminis T. Thomas M. S. C. (2014). Bilingual language switching in emmo production: empirical and computational studies. *Biling. Lang. Cogn.* 17 294–315.

[B27] Finkbeiner M. Gollan T. H. Caramazza A. (2006). Lexical access in bilingual speakers: What’s the (hard) problem? *Biling. Lang. Cogn.* 9 153–166.

[B28] Forster K. I. Davis D. (1984). Repetition priming and frequency attenuation in lexical access. *J. Exp. Psychol. Learn. Mem. Cogn.* 10 680–698.

[B29] Gollan T. H. Sandoval T. Salmon D. P. (2011). Cross-language intrusion errors in aging bilinguals reveal the link between executive control and language selection. *Psychol. Sci.* 22 1155–1164. 10.1177/0956797611417002 21775653 PMC3598590

[B30] Grainger J. Beauvillain C. (1987). Language blocking and lexical access in bilinguals. *Q. J. Exp. Psychol.* 39 295–319.

[B31] Grainger J. Dijkstra T. (1992). “On the representation and use of language information in bilinguals,” in *Cognitive Processing in Bilinguals*, ed. Harris R. J. (Amsterdam: North Holland), 207–220.

[B32] Green D. W. (1998). Mental control of the bilingual lexico-semantic system. *Biling. Lang. Cogn.* 1 213–229.

[B33] Hermans D. Bongaerts T. de Bot K. Schreuder R. (1998). Producing words in a foreign language: Can speakers prevent interference from their first language? *Biling. Lang. Cogn.* 1 213–229.

[B34] Hernandez A. E. Kohnert K. J. (1999). Aging and language switching in bilinguals. *Aging Neuropsychol. Cogn.* 6 69–83. 10.1076/anec.6.2.69.783

[B35] Hirsch P. Declerck M. Koch I. (2015). Exploring the functional locus of language switching: evidence from a PRP paradigm. *Acta Psychol.* 116 1–6. 10.1016/j.actpsy.2015.07.010 26280496

[B36] Jackson G. M. Swainson R. Cunnington R. Jackson S. R. (2001). ERP correlates of executive control during repeated language switching. *Biling. Lang. Cogn.* 4 169–178.

[B37] Jaeger T. F. (2008). Categorical data analysis: away from ANOVAs (transformation or not) and towards logit mixed models. *J. Mem. Lang.* 59 434–446. 10.1016/j.jml.2007.11.007 19884961 PMC2613284

[B38] Kass R. E. Raftery A. E. (1995). Bayes factors. *J. Am. Stat. Assoc.* 90 773–795.

[B39] Kroll J. Tokowicz N. (2001). “The development of conceptual representation for words in a second language,” in *One Mind, Two Languages: Bilingual Language Processing*, ed. Nicol J. (Malden, MA: Blackwell), 49–71.

[B40] Kroll J. Tokowicz N. (2005). “Models of bilingual representation and processing,” in *Handbook of Biling.: Psycholinguistic Approaches*, eds Kroll J. F. De Groot A. M. B. (Oxford: Oxford University Press), 531–553.

[B41] Kroll J. F. Dijkstra A. (2002). “The bilingual lexicon,” in *Handbook of Applied Linguistics*, ed. Kaplan R. (Oxford: Oxford University Press), 301–321.

[B42] Kroll J. F. Gullifer J. W. Rossi E. E. (2013). The multilingual lexicon: the cognitive and neural basis of lexical comprehension and production in two or more languages. *Annu. Rev. Appl. Linguist.* 33 102–127.

[B43] Kroll J. F. Stewart E. (1994). Category interference in translation and picture naming: evidence for asymmetric connections between bilingual memory representations. *J. Mem. Lang.* 33 149–174. 10.1016/j.jecp.2008.10.004 19084237

[B44] Lemhöfer K. Broersma M. (2012). Introducing LexTALE: a quick and valid lexical test for advanced learners of English. *Behav. Res. Methods* 44 325–343. 10.3758/s13428-011-0146-0 21898159 PMC3356522

[B45] Lemhöfer K. Dijkstra T. (2004). Recognizing cognates and interlingual homographs: effects of code similarity in language-specific and generalized lexical decision. *Mem. Cogn.* 32 533–550. 15478748 10.3758/bf03195845

[B46] Lemhöfer K. Dijkstra T. Michel M. (2004). Three languages, one ECHO: cognate effects in trilingual word recognition. *Lang. Cogn. Process.* 19 585–611.

[B47] Lemhöfer K. Dijkstra T. Schriefers H. Baayen R. H. Grainger J. Zwitserlood P. (2008). Native language influences on word recognition in a second language: a megastudy. *J. Exp. Psychol. Learn. Mem. Cogn.* 34 12–31. 10.1037/0278-7393.34.1.12 18194052

[B48] Macizo P. Bajo T. Paolieri D. (2012). Language switching and language competition. *Second Lang. Res.* 28 131–149.

[B49] Meuter R. F. I. Allport A. (1999). Bilingual language switching in naming: asymmetrical costs of language selection. *J. Mem. Lang.* 40 25–40. 10.1006/jmla.1998.2602 23957363

[B50] Midgley K. J. Holcomb P. J. Vanheuven W. J. Grainger J. (2008). An electrophysiological investigation of cross-language effects of orthographic neighborhood. *Brain Res.* 1246 123–135. 18948089 10.1016/j.brainres.2008.09.078 PMC2656968

[B51] Mosca M. de Bot K. (2017). Bilingual language switching: production vs. recognition. *Front. Psychol.* 8:934. 10.3389/fpsyg.2017.00934 28638361 PMC5461355

[B52] Olson D. J. (2016). Bilingual language switching costs in auditory comprehension. *Lang. Cogn. Neurosci.* 32 494–513. 10.1080/23273798.2016.1250927

[B53] Orfanidou E. Sumner P. (2005). Language switching and the effects of orthographic specificity and response repetition. *Mem. Cogn.* 33 355–369. 16028589 10.3758/bf03195323

[B54] Philipp A. M. Gade M. Koch I. (2007). Inhibitory processes in language switching: evidence from switching language-defined response sets. *Eur. J. Cogn. Psychol.* 19 395–416.

[B55] Philipp A. M. Huestegge L. (2015). Language switching between sentences in reading: exogenous and endogenous effects on eye movements and comprehension. *Biling. Lang. Cogn.* 18 614–625.

[B56] Philipp A. M. Koch I. (2009). Inhibition in language switching: What is inhibited when switching between languages in naming tasks? *J. Exp. Psychol. Learn. Mem. Cogn.* 35 1187–1195. 10.1037/a0016376 19686014

[B57] Proverbio A. M. Lenio G. Zani A. (2004). Language switching mechanisms in simultaneous interpreters: an ERP study. *Neuropsychologia* 42 1636–1656. 10.1016/j.neuropsychologia.2004.04.013 15327931

[B58] Rouder J. N. Speckman P. L. Sun D. Morey R. D. Iverson G. (2009). Bayesian t tests for accepting and rejecting the null hypothesis. *Psychon. Bull. Rev.* 16 225–237. 10.3758/PBR.16.2.225 19293088

[B59] Slevc L. R. Davey N. S. Linck J. A. (2016). A new look at “the hard problem” of bilingual lexical access: evidence for language-switch costs with univalent stimuli. *J. Cogn. Psychol.* 28 385–395.

[B60] Struys E. Woumans E. Nour S. Kepinska O. Van den Noort M. (2019). A domain-general monitoring account of language switching in recognition tasks: evidence for adaptive control. *Biling. Lang. Cogn.* 22 606–623.

[B61] Szubko-Sitarek W. (2011). Cognate facilitation effects in trilingual word recognition. *Stud. Second Lang.* 1 189–208.

[B62] Thomas M. S. C. Allport A. (2000). Language switching costs in bilingual visual word recognition. *J. Mem. Lang.* 43 44–66. 10.1037/a0034060 23957363

[B63] van Heuven W. J. B. Dijkstra T. Grainger J. (1998). Orthographic neighborhood effects in bilingual word recognition. *J. Mem. Lang.* 39 458–483.

[B64] van Kesteren R. Dijkstra T. de Smedt K. (2012). Markedness effects in Norwegian–English bilinguals: task-dependent use of language-specific letters and bigrams. *Q. J. Exp. Psychol.* 65 2129–2154. 10.1080/17470218.2012.679946 22554207

[B65] von Studnitz R. E. Green D. W. (2002). Interlingual homograph interference in German-English bilinguals: Its modulation and locus of control. *Biling. Lang. Cogn.* 5 1–23.

[B66] Wu Y. J. Thierry G. (2010). Chinese–english bilinguals reading English hear Chinese. *J. Neurosci.* 30 7646–7651. 20519539 10.1523/JNEUROSCI.1602-10.2010 PMC6632379

