# Supplementary material for: The Effect of the Non-task Language When Trilingual People Use Two Languages in a Language Switching Experiment
Source: Front Psychol. 2020 Apr 30;11:754. doi: 10.3389/fpsyg.2020.00754 (PMC7204993; doi:10.3389/fpsyg.2020.00754)
Supplement: Supplementary file 1 [file Data_Sheet_1.docx]

**Appendix A**

30 L2 Chinese words

| 笑 书 旗 图画 家乡 戒指 西红柿 艺术家 树袋熊 女演员  诗 光 美 农民 态度 建筑 建议 博物馆 警察局 动物园  心 闻 家 考试 友谊 河流 主人 在室内 俱乐部 电视机 |
| --- |

30 L2 Chinese non-words

| 阅渎 炼习 跑布 考式 期沫 班马线 由菜花 游永池 天安们 大羊洲  安全代 穷光旦 跳望 木浴 宁檬 儿童结 块乐 美俪 花园 一吓子  图书管 身国旗 保户 甘楠 芽齿 走璐 命跟子 磨术师 电疯扇 鹏友 |
| --- |

30 L1 Tibetan words


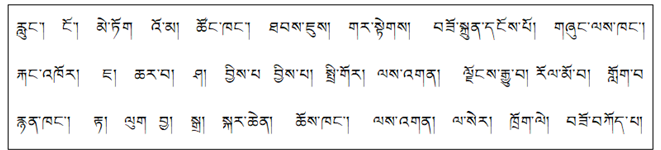


30 L1 Tibetan non-words


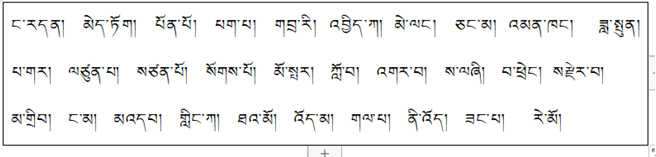


**Appendix B**

30 L1 Tibetan words


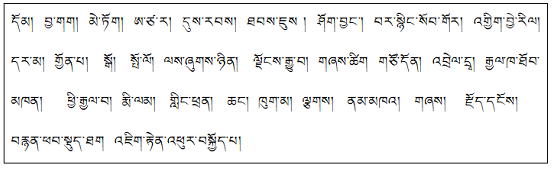


30 L1 Tibetan non-words


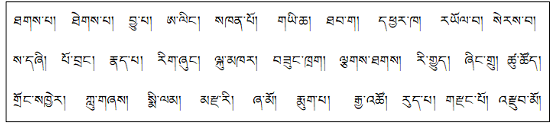


30 L3 English words

| father face cat chicken brain city clock cabbage driver hamburger  ice snow paper sky pen moon arm grandparent Christmas England  cow pig bed money clothes nose tiger robot holiday computer |
| --- |

30 L3 English non-words

| ssight peoppple catchh quickk wwrite Chinaa mixx llife thanksk finishh  kiddd wwrong nearbby computor affter rrather listem hearr thaaat foodd  rright illl difcult eachch awway neeed lllong helloo ggame ouuut |
| --- |

**Appendix C**

30 L2 Chinese words

| 风 脸 花 牛奶 晚会 商店 舞台 建筑物 办公室 自行车  茶 雨 肉 孩子 手机 蛋糕 作者 旅行者 牛仔裤 电影院  马 羊 鸟 声音 明星 教堂 任务 胡萝卜 巧克力 工程师 |
| --- |

30 L2 Chinese non-words

| 虹绿灯 记念日 伙龙果 占斗机 藏玲羊 和坪 粗广 打蕾 萍果 薯假  即然 气车 正据 篮天 坐位 库子 澳运会 百和花 解方军 猫头应  打驾 眼竟 吉详 错吴 礼冒 草评 鱼尾文 练习提 极时雨 记亿力 |
| --- |

30 L3 English words

| laugh book flag picture homeland ring tomato artist koala actress  poem light beauty farmer attitude building suggestion museum police station zoo  heart smell home exam friendship river owner indoors club television |
| --- |

30 English non-words

| aapple lookk llove dayww fulll tturn yelloww evvven sounddd manymm  morrre interestin buyyy ooour goood howw fuun happyy timee exxist  offf cheerr ppay dancee deeaal noww ssimple dowwn rooom lettt |
| --- |
